# Supplementary material for: Sarcopenic Obesity Is a Risk Factor for Worse Oncological Long-Term Outcome in Locally Advanced Rectal Cancer Patients: A Retrospective Single-Center Cohort Study
Source: Nutrients. 2023 Jun 5;15(11):2632. doi: 10.3390/nu15112632 (PMC10255819; doi:10.3390/nu15112632)
Supplement: Supplementary file 1 [file nutrients-15-02632-s001.zip › Supplement Material/Supplement Figure 1.pdf]

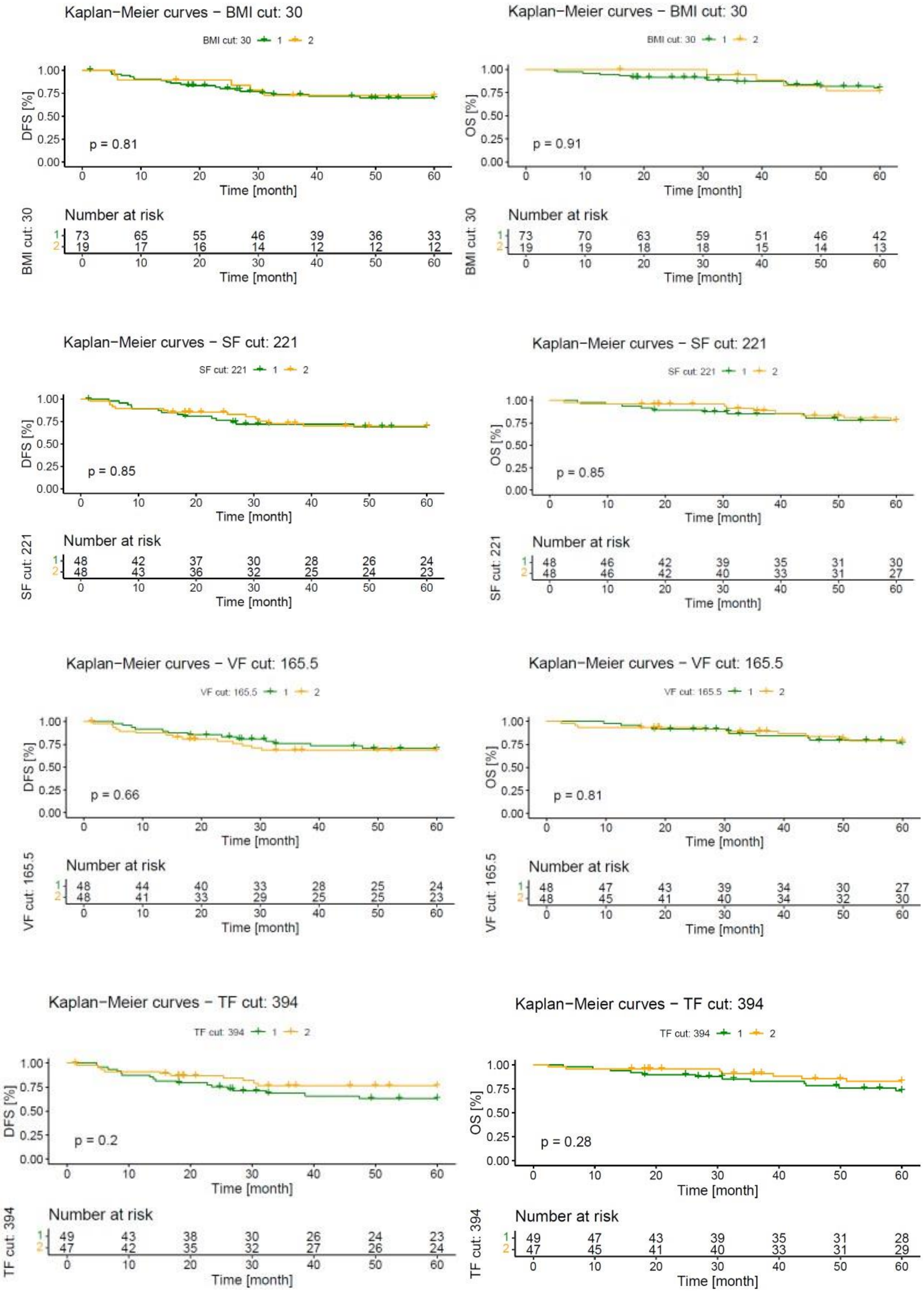

Supplement Figure 1: Kaplan-Meier survival curves of several body composition indexes. (BMI, Subcutaneous fat, Visceral fat, Total fat). Abbreviations: BMI = Body mass index, SF = Subcutaneous fat, VF = Visceral fat, TF = Total fat, DFS = Disease free survival, OS = Overall survival.
