# Supplementary material for: Sarcopenic Obesity Is a Risk Factor for Worse Oncological Long-Term Outcome in Locally Advanced Rectal Cancer Patients: A Retrospective Single-Center Cohort Study
Source: Nutrients. 2023 Jun 5;15(11):2632. doi: 10.3390/nu15112632 (PMC10255819; doi:10.3390/nu15112632)
Supplement: Supplementary file 1 [file nutrients-15-02632-s001.zip › Supplement Material/Supplement Table 1.pdf]

Supplement Table 1: Cut off levels of body composition indexes		
	Male	Female
Total fat (cm^2)	>495	>392
Visceral fat (cm^2)	>163,8	>80,1
Subcutaneous fat (cm^2)	>210	>274
VF/TF ratio	>0,397	>0,330
SF/TF ratio	>0,496	>0,715
SMA (cm^2)	<134	<89,2
SMI (cm^2/m^2)	<41,6	<32
Sarcopenic obesity	Low SMA / high VF	

Abbreviations: VF = Visceral fat, TF = Total fat, SF = Subcutaneous fat, SMA = Skeletal muscle area, SMI = Skeletal muscle index.
